# Correlation of the *DRD2* gene polymorphism with psychopathology and predictive antimanic responses in patients with bipolar mania

**DOI:** 10.3389/fphar.2024.1465356

**Published:** 2024-11-26

**Authors:** Hejian Tao, Haiying Jin, Min Xu, Haihan Chen, Fengli Sun, Weidong Jin

**Affiliations:** ^1^ Department of Psychiatry, Jinhua Municipal Center Hospital, Jinhua, China; ^2^ Department of Psychiatry, Zhejiang Chinese Medical University, Hangzhou, China; ^3^ Department of Psychiatry, Hospital of Zhejiang Province, Hangzhou, China; ^4^ Department of Psychiatry, Zhejiang Province Mental Health Center, Hangzhou, China

**Keywords:** bipolar mania, DRD2 gene, gene polymorphism, lithium, olanzapine

## Abstract

To explore the correlation of the *DRD2* gene polymorphism with psychopathology and predict responses in patients with mania treated with lithium and olanzapine. Sixty patients with bipolar mania were treated with lithium combined with olanzapine for 8 weeks and assessed using YMRS, HAMD, and HAMA. The *DRD2* gene polymorphism rs1800497 was tested. Eleven (24.4%) manic patients achieved an early effective response according to the reduction of the YMRS score of >20% in the 2nd week, with a lower HAMA score than the no early effective response group. Twenty-three (51.1%) manic patients achieved remission according to the reduction of the YMRS score of >75% at the 8th week with a higher dose of lithium at the 8th weekend (g/day) than in the no-remission group. Manic patients with genotype GG had lower YMRS scores and lower doses and serum concentrations of olanzapine than patients with genotype AA + AG from the 4th week to the 8th week. Manic patients with genotype GG had a higher relative change in the YMRS score than those with genotype AA + AG from the 2nd week to the 8th week. No differences in HAMA or HAMD were found between the groups with genotype GG and AA + AG. There were more patients who achieved an early effective response in the 2nd week and remission in the 8th in those with genotype GG compared to those with genotype AA + AG. Manic patients with genotype GG had a greater improvement in the YMRS score due to a greater early effective response and remission, which was not related to higher doses and serum concentrations of olanzapine and lithium.

## 1 Introduction

Bipolar affective disorder is a common neuropsychiatric disorder. Although its neurobiological underpinnings are incompletely understood, the dopamine hypothesis has been a key theory of the pathophysiology of both manic and depressive phases of the disease for more than four decades (Ashok et al., 2017). Over the years, many genetic polymorphisms have been identified as having a greater risk of developing mood disorders. The countries with the highest activity and the most impactful research in the field were identified. Furthermore, a total of 13 main thematic clusters emerged in the literature. From the qualitative inspection of the clusters, it emerged that the research interest moved from a monogenic to a polygenic risk framework. Researchers have moved from studying single genes in the early 1990s to conducting genome-wide association studies around 2015 (Bonacina et al., 2023). The increased use of antidopaminergics in the treatment of this disorder and new *in vivo* neuroimaging and postmortem studies make it timely to review this theory of dopamine hypothesis. The possible influence of dopamine receptor variants on drug response has not received as much attention. In contrast, there is some evidence that polymorphisms and mutations in dopamine receptors can alter functional activity and pharmacological profiles, but no conclusive data link these gene variants to drug response or disease. The lack of unequivocal findings may be related, in part, to the subtle changes in receptor pharmacology that these polymorphisms and mutations mediate. These subtle effects may be obscured by the influence of genes controlling drug metabolism and kinetics (Wong et al., 2000).

In a systematic review, most antipsychotics, carbamazepine, lithium, tamoxifen, and valproate, were effective for acute mania, although only aripiprazole, olanzapine, quetiapine, and risperidone had better acceptability than placebo (Kishi et al., 2022). In particular, cariprazine (CAR) is an antipsychotic drug for the treatment of schizophrenia and bipolar disorder (BD) and acts as a partial agonist on dopamine receptors (DR), D2, and D3. The study found a significant association between *DRD2* rs1800497 and rs6277 and the response to Cariprazine (CAR) treatment (De Pieri et al., 2023). When genotypes were combined to obtain an arbitrary score, the receiver operating characteristic curve analysis showed, for the first time, a correlation between single nucleotide polymorphism (SNPs) in *DRD2* and the response to CAR treatment. In several studies, *DRD2* gene polymorphism was related to olanzapine effectiveness and safety variability (Zubiaur et al., 2021). These observations suggest that antipsychotics, such as risperidone and aripiprazole, can lead to the deposition of long-lasting epigenetic marks in addition to interacting with specific receptors, impairing the function of the nervous system (Osuna-Luque et al., 2018). There is reason to believe that the efficacy of olanzapine as an atypical antipsychotic in the treatment of mania is related to dopamine D2 receptor polymorphism.

This study aimed to find an association between *DRD2* rs1800497 and olanzapine combined with lithium in the treatment of patients with bipolar mania.

## 2 Materials and methods

### 2.1 Research object

Patients with mania hospitalized at Tongde Hospital in Zhejiang Province, Jiaxing Kangci Hospital, Shaoxing Seventh Hospital, and Jinhua Second Hospital from 31 December 2020, to 1 January 2022, were selected according to the Diagnostic and Statistical Manual of Mental Disorders, Fifth Edition.

### 2.2 Research methods

Blood sample collection, DNA extraction, and gene polymorphism monitoring: 5 mL of peripheral venous blood was collected, and anticoagulated with ethylenediaminetetraacetic acid was also stored at −20°C. DNA extraction kit (TSINGKE) was used to extract genomic DNA, and the nucleic acid protein analyzer monitors its concentration and purity. A polymerase chain reaction was used to amplify the target gene containing the *DRD2* gene rs1800497 polymorphism. The primer was designed and synthesized, the sequence of the SNP site was found at NCBI (https://www.ncbi.nlm.nih.gov/), and the peripheral amplification primer was designed. The guiding primer and folding primer of rs1800497 were ATCCTCAAAGTGCTGGTC and AGG​CAG​GCG​CCC​AGC​TGG, respectively. The amplification product was digested at 65°C by restriction endonuclease for 3 h. The genotype of the enzyme product was identified by 3% agarose gel electrophoresis and stained with bromophenol blue.

### 2.3 Drugs and management

The patients were treated with lithium carbonate combined with olanzapine from baseline for 8 weeks. The dose of lithium carbonate was 0.9–1.2 g/day and the dose of olanzapine was 15–25 mg/day. Other psychotropic drugs were not used in combination during treatment. If adverse reactions occur, benzodiazepines are administered in combination for symptomatic treatment.

### 2.4 Clinical assessment

#### 2.4.1 Scale assessment

Young manic rating scale (YMRS), Hamilton Anxiety Scale (HAMA), and Hamilton Depression Scale (HAMD) were used to assess clinical symptoms and changes by two psychiatrists on the 2nd, 4th, 6th, and 8th weekends before and after treatment with consistency ≥80%.

#### 2.4.2 Clinical efficacy

The main efficacy was defined as a decrease in the level of evaluation of YMRS. YMRS reduction score % = (total score before treatment - total score after treatment)/(total score before treatment) × 100%. Early effective therapy was expressed as an early effective response rate, which means that the YMRS reduction rate >20% on the 2nd week. Remission was expressed as a remission rate, which means a YMRS reduction rate ≥75% on the 8th week (Jin et al., 2003).

### 2.5 Statistics analysis

Statistical analysis of the data was performed using SPSS 22.0 software. Measurement data were presented as mean ± standard deviation, and an independent sample *t*-test was performed to compare differences between the two groups. The counting data were presented as composition ratio or frequency, and the chi-square test was used for comparison between groups. The statistical significance was set at P < 0.05.

**FIGURE 1:**
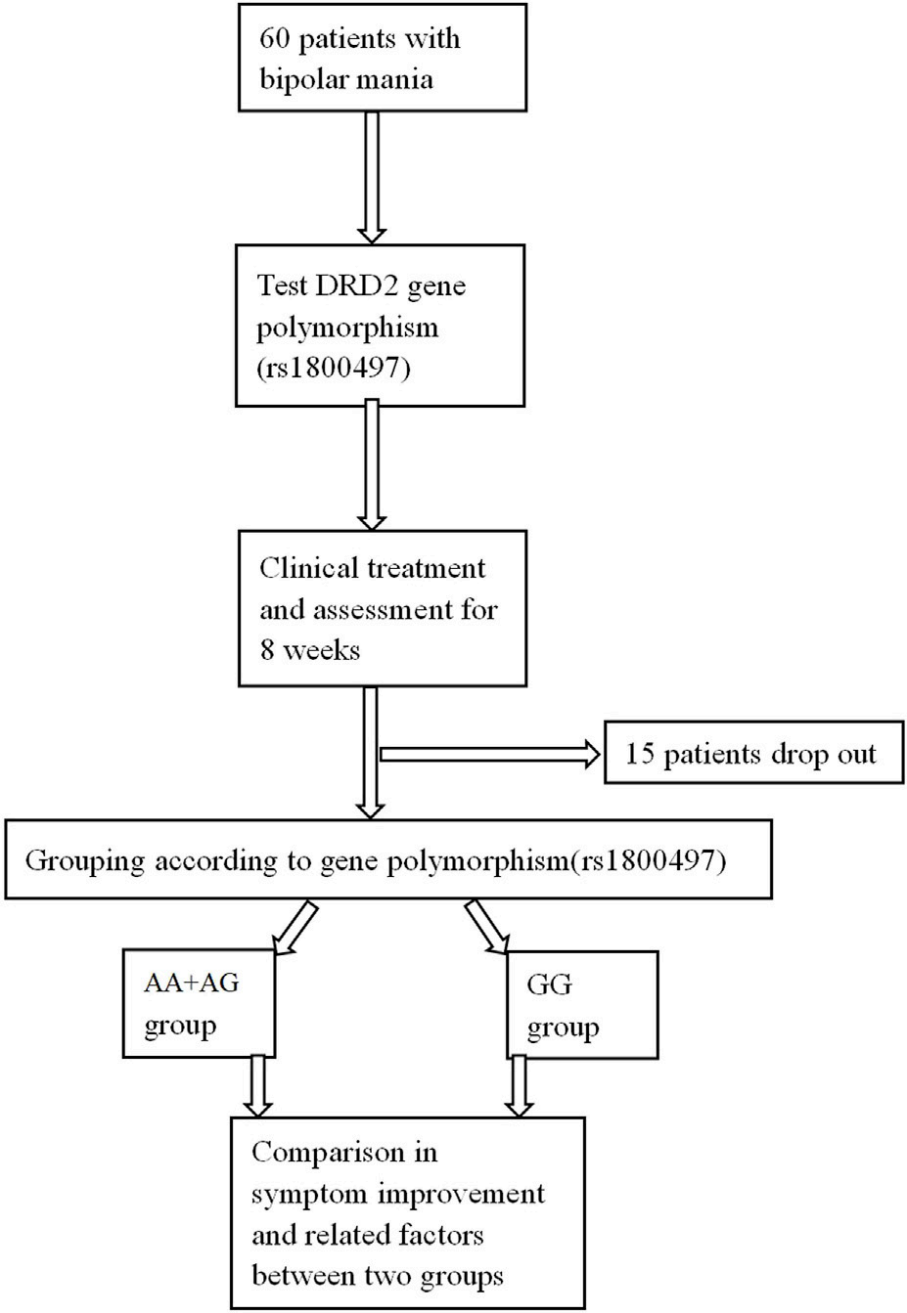
Research flowchart.

This study was approved by the Medical Ethics Committee of Tongde Hospital of Zhejiang Province (2019–070) and registered for clinical research in China (CHICT2100054696). The research flowchart is shown in Figure 1.

## 3 Results

### 3.1 Comparison of relative factors of patients and their therapeutic efficacy

We evaluated 45 patients with bipolar mania and observed changes in their YRMS score (reduction of >20% in the 2nd week and 75% in the 8th week) after 8 weeks of treatment with lithium combined with olanzapine. According to the reduction in the YRMS score, 11 patients were assigned to the early effective response group, with a reduction of a YMRS score >20% in the 2nd week, and 34 patients to the no early effective response group, with a reduction ≤20% in the 2nd week. No differences in sex, age, YMRS, HAMD, HAMA, drug dose, and concentration were found between the two groups, except for a relative reduction in YMRS in the 2nd week (see Table 1).

**TABLE 1:** The relative factors of 45 manic patients, early effective response group and no-early effective response group.

Item	All	Early effective response group	No early effective response group	*X* ^*2*^ */t*	*P*
Number of cases	45	11 (24.4%)	34 (75.6%)		
Age	32.3 ± 9.9	32.1 ± 9.5	32.3 ± 10.2	0.057	0.953
Age at first onset	32.3 ± 9.9	21.6 ± 6.9	24.1 ± 9.9	0.758	0.374
Sex (M/F)	21/24	5/6	16/18		
Dosage of olanzapine at 2nd weekend (mg/d)	11.5 ± 2.9	11.8 ± 2.5	11.4 ± 3.1	0.333	0.713
Dosage of lithium at 2nd weekend (g/d)	0.8 ± 0.2	0.8 ± 0.1	0.7 ± 0.2	0.593	0.526
Concentration of olanzapine at 2nd weekend (ng/mL)	36.2 ± 16.6	39.4 ± 18.6	35.1 ± 16.1	0.726	0.472
Concentration of lithium at 2nd weekend (mmol/L)	0.6 ± 0.1	0.6 ± 0.08	0.6 ± 0.1	0.622	0.537
YMRS at 2nd weekend	25.9 ± 2.6	25.5 ± 3.6	26.0 ± 2.2	0.527	0.601
HAMD at 2nd weekend	6.6 ± 1.1	6.3 ± 0.8	6.7 ± 1.2	0.824	0.308
HAMA at 2nd weekend	11.3 ± 1.3	10.6 ± 1.2	11.5 ± 1.3	2.081	0.049
Relative reduction of YMRS at 2nd weekend (%)	15.7 ± 6.7	25.3 ± 3.1	12.6 ± 3.7	10.199	0.000

Patients with a reduction of YMRS score ≥75% in the 8th week were assigned to the remission group; meanwhile, 22 patients with a reduction of YMRS score <75% in the 8th week were in the no-remission group. There were no differences in sex, age, HAMD, HAMA, drug dosage, and concentration of olanzapine between the two groups. The difference in dose of lithium, YMRS, and relative reduction of YMRS at the 8th week between the two groups was found (see Table 2).

**TABLE 2:** The relative factors of 45 manic patients, remission group and no-remission group.

Item	All	Remission group	No remission group	*X* ^*2*^ */t*	*P*
Number of cases	45	23 (51.1%)	22 (48.9%)		
Age	32.3 ± 9.9	33.8 ± 10.6	30.7 ± 9.1	1.058	0.294
Age at first onset	23.4 ± 9.2	23.3 ± 8.4	23.5 ± 10.2	0.071	0.943
Sex (M/F)	21/24	12/11	9/13		
Dosage of olanzapine at 8th weekend (mg/d)	13.9 ± 2.6	14.3 ± 2.7	13.5 ± 2.5	1.050	0.299
Dosage of lithium at 8th weekend (g/d)	0.8 ± 0.1	0.8 ± 0.1	0.7 ± 0.1	2.247	0.003
Concentration of olanzapine at 8th weekend (ng/mL)	64.4 ± 18.0	69.3 ± 19.5	59.3 ± 15.2	1.896	0.054
Concentration of lithium at 8th weekend (mmol/L)	0.7 ± 0.1	0.7 ± 0.2	0.6 ± 0.1	1.985	0.052
YMRS at 8th weekend	8.0 ± 1.2	7.1 ± 1.0	8.8 ± 0.8	5.740	0.000
HAMD at 8th weekend	10.5 ± 0.6	10.9 ± 0.6	10.5 ± 0.5	0.857	0.380
HAMA at 8th weekend	6.3 ± 1.4	6.7 ± 1.5	6.4 ± 1.2	0.890	0.379
Relative reduction of YMRS at 8th weekend (%)	73.8 ± 4.9	77.8 ± 2.3	69.9 ± 2.9	10.407	0.000

### 3.2 Therapeutic efficacy and *DRD2* gene polymorphism of rs1800497

The *DRD2* gene polymorphism of rs1800497 in 45 patients with mania distributes 17 GG, 20 GA, and 8 AA. A previous study showed that the G allele may be related to more responses (De Pieri et al., 2023). We compare the relative factors between the genotype GG groups and genotype AA + AG groups. There were no differences in sex, age, marriage, education, family history, clinical type, and dose and concentration of lithium between the two groups, except for the dose and concentration of olanzapine (see Table 3).

**TABLE 3:** Primary information between different groups of rs1800497 (AA + AG and GG genotype).

Item	information	AA + AG	GG	*X* ^2^	*P*
Sex	Male	13	8	0.002	0.967
	Female	15	9		
Age	17–30	10	8	0.640	0.726
	31–40	11	5		
	41–60	7	4		
Marriage	Unmarried	11	11	2.917	0.233
	Married	13	4		
	Divorce	4	2		
Education	Primary school	0	1	5.578	0.233
	Junior school	3	1		
	Senior school	5	7		
	Junior college	8	2		
	University	12	6		
Age of first onset	Less 17	7	5	3.876	0.275
	17–30	18	9		
	31–40	1	3		
	41–60	2	0		
Family history	Positive	2	3	1.182	0.277
	Negative	26	14		
Clinical type	I type	18	7	2.522	0.283
	II type	6	3		
	Mixed episode	10	7		
Dosage of medicine	Olanzapine (mg/d)	14.55 ± 2.81	12.95 ± 2.02	2.060	0.045
	Lithium carbonate (g/d)	0.84 ± 0.15	0.78 ± 0.15	1.280	0.208
Concentration of medicine	Olanzapine (ng/mL)	68.68 ± 18.58	57.51 ± 15.18	2.088	0.043
	Lithium carbonate (mmol/L)	0.70 ± 0.13	0.67 ± 0.17	0.627	0.543

The difference in YMRS and the relative change between the genotype GG groups and AA + AG groups were analyzed. The difference in YMRS appeared in the 4th week, and the relative change in YMRS appeared in the 2nd week between the genotype GG groups and genotype AA + AG groups (see Table 4).

**TABLE 4:** Symptom and Change between different between groups of rs1800497 (AA + AG and GG genotype).

Scale/Relative change	Weeks	Group		*t*	*P*_1_	F	*P*_2_
		AA + AG ( $\bar{x}$ ±s)	GG ( $\bar{x}$ ±s)				
YMRS	Week 0	30.64 ± 3.71	31.35 ± 4.24	−0.59	0.56	2.580	0.115
	Weekend 2	26.29 ± 3.00	25.29 ± 1.76	1.40	0.17		
	Weekend 4	20.86 ± 2.86	19.24 ± 2.02	2.05	0.05		
	Weekend 6	15.79 ± 3.01	13.24 ± 2.17	3.04	0.00		
	Weekend 8	8.39 ± 1.34	7.35 ± 0.93	2.80	0.00		
Relative change of YMRS (%)	Week 0	—	—	—	—		
	Weekend 2	13.96 ± 6.46	18.63 ± 5.90	−2.43	0.02	13.625	0.001
	Weekend 4	31.93 ± 4.77	38.11 ± 6.66	−3.62	0.00		
	Weekend 6	48.33 ± 9.24	57.53 ± 6.26	−3.63	0.00		
	Weekend 8	72.35 ± 5.04	76.27 ± 3.76	−2.76	0.00		
HAMD	Week 0	6,29 ± 1.51	6 ± 1.23	−0.66	0.51	1.137	0.292
	Weekend 2	6.55 ± 1.30	6.41 ± 1.00	−0.92	0.36		
	Weekend 4	8.145 ± 1.48	7.71 ± 0.92	1.22	0.23		
	Weekend 6	9.18 ± 1.25	9.47 ± 0.87	−0.85	0.36		
	Weekend 8	10.54 ± 0.58	10.47 ± 0.62	0.36	0.72		
HAMA	Week 0	13.89 ± 1.69	13 ± 1.22	1.97	0.06	0.903	0.347
	Weekend 2	13.36 ± 1.77	13.41 ± 1.00	−0.30	0.77		
	Weekend 4	13.72 ± 2.63	12.94 ± 3.03	−0.93	0.36		
	Weekend 6	14.82 ± 3.18	15.53 ± 2.79	−0.05	0.96		
	Weekend 8	15.43 ± 3.814	16.29 ± 3.37	−0.31	0.76		

The correlation between therapeutic efficacy and gene polymorphism was also found. The early response in the 2nd week correlates with genotype GG (see Table 5). The higher early response in the 2nd week in patients with genotype GG of *DRD2* gene polymorphism rs1800497 was found than that in patients with genotype AA + AG. Remission in the 8th week also correlates with genotyp GG (see Table 6). The higher remission in the 8th week in patients with genotype GG of *DRD2* gene polymorphism rs1800497 was found than that in patients with genotype AA + AG.

**TABLE 5:** Comparison of response at second weekend between groups of rs1800497 (AA + AG and GG genotype).

		response at 2nd week		Total
		≤20%	>20%	
RS1800497	AA + AG	24	4	28
	GG	10	7	17
Total		34	11	45
*X* ^*2*^ = 4.141 *p* = 0.042				

**TABLE 6:** Comparison of remission at eighth weekend between groups of rs1800497 (AA + AG and GG genotype).

		remission at 8th week		Total
		<75%	≥75%	
RS1800497	AA + AG	18	10	28
	GG	4	13	17
Total		22	23	45
*X* ^2^ = 7.032 *p* = 0.001				

## 4 Discussion

Atypical antipsychotics, such as olanzapine combined with mood stabilizers, such as lithium carbonate, are commonly used and effective treatment methods for mania. Clinical studies and evidence-based medicine research have shown that olanzapine and lithium carbonate are effective drugs for treating mania. Treatment also suggest that the combination of lithium carbonate and olanzapine may yield better resultsfor treatment of mania (Jin et al., 2004; McKnight et al., 2019; Chen and Jin, 2022; Yatham et al., 2021; Tang et al., 2007).Therefore, by the combination of lithium and olanzapine as a therapeutic treatment, 11 (24.4%) of the patients achieved early effective response in the 2nd week, and 23 (51.1%) achieved remission in the 8th week of 8-week treatment, which certainly suggested that the combination of lithium and olanzapine showed more improvement in manic patients (see Tables 1, 2). Another finding of the study was that the remission group had higher doses of lithium carbonate, as well as possibly higher concentrations of lithium carbonate and olanzapine, which were clearly consistent with clinical practice (Table 2).

However, the next analysis had different results, which differed from the correlation between good efficacy and high doses or concentrations of the drug. The results show that patients with AA + AG of the *DRD2* gene polymorphism have a higher dose and concentration of olanzapine than those with AA of the *DRD2* gene polymorphism (Table 3), but patients with AA + AG of the *DRD2* gene polymorphism achieve a greater improvement in symptoms compared to those with AA of the *DRD2* gene polymorphism, which concludes a greater relative reduction in manic symptoms (Table 4), a higher early response rate and remission in the 2nd (Table 5) and 8th week (Table 6), respectively. These results suggest that therapeutic effects are related to the *DRD2* gene polymorphism of rs1800497 rather than the higher dose and concentration of the drug. Personalized precision therapy should focus on polymorphisms, not drug concentrations or doses, although drug metabolism and concentration are also involved in the polymorphism of certain metabolic enzymes (Milosavljevic et al., 2021). It also suggests that pharmacodynamics is very important in personalized precision therapy, at least in the treatment of mania (De Pieri et al., 2023; Urs et al., 2012). This also appeared to confirm the dopamine hypothesis that manic episodes are associated with dopamine hyperactivity, with dopamine receptor polymorphism more closely (Bonacina et al., 2023; Wong et al., 2000; Azechi et al., 2019).

Another finding of this study is equally significant since patients with genotype GG have early and late good treatment results, which appears to validate that “early effective response predicts late efficacy.” Early improvement in HAMD-17 and HAMD-6 scores was found to predict ultimate response and remission in depressed patients treated with fluoxetine or ECT (Lin and Lin, 2019). Moreover, early improvement with vortioxetine was also found to predict response and remission in depressive patients (Inoue et al., 2021). This prediction pattern occurs not only in the treatment of depression, but in fact in the treatment of mania. Response and remission could be predicted by early improvement at week 2, while patients without early improvement were unlikely to reach response and remission at week 4 (Li et al., 2017). Early response at week 1 can predict treatment outcomes in adolescents with bipolar mania or mixed episodes treated with olanzapine (Xiao et al., 2017). We also found that an early improvement in more manic symptoms in the genotype GG also suggested a higher rate of remission. Therefore, it is reasonable to assume that this predictive pattern is also present in the treatment of patients with mania and that this predictive pattern is mediated by the gene polymorphism of rs1800497 with GG.

The shortcomings of this study are as follows. First, as a genetics and genetic polymorphism study, the sample size of the above studies is still small, and larger sample sizes and multi-center studies may lead to more reliable conclusions. Second, the treatment we used was a combination of lithium carbonate and olanzapine, making it difficult to distinguish between the therapeutic effects, which belong to lithium or olanzapine. It should be noted that combination therapy is more effective than monotherapy, especially lithium carbonate plus olanzapine, in the treatment of mania and even for refractory mania (Fountoulakiskn et al., 2022; Tohen et al., 2002). Third, the scale of the study does not involve psychotic symptoms because these psychotic symptoms are relatively common in mania and are also important for therapeutic intervention (Bjørklund et al., 2017). Fourth, dopamine gene polymorphism can be involved in multiple sites, and we only chose rs1800497, but there may also be another gene that shares this characteristic.

## Data Availability

The original contributions presented in the study can be found here: https://db.cngb.org/search/project/CNP0006542/. Further inquiries can be directed to the corresponding author.
